# The association between pregnancy levels of blood lipids and the risk of preterm birth

**DOI:** 10.1038/s41598-024-61119-x

**Published:** 2024-05-11

**Authors:** Yao Lv, Liang Xu, Zhong He, Xiaorui Liu, Yuna Guo

**Affiliations:** 1 The International Peace Maternity and Child Health Hospital, School of Medicine, Shanghai Jiao Tong University, Hengshan Road 910, Shanghai, 200030 China; 2 Shanghai Key Laboratory of Embryo Original Diseases, Shanghai, China; 3 Shanghai Municipal Key Clinical Specialty, Shanghai, China; 4 Songjiang Maternity and Child Health Hospital, Shanghai, China

**Keywords:** Hyperlipidemia, Restricted cubic spline, Hypertriglyceridemia, Hyperlipidemia in pregnancy, Preterm delivery, Predictive markers, Risk factors

## Abstract

Preterm labor, a condition associated with various risk factors such as a history of prior preterm birth (PTB) and multiple pregnancies, has recently seen an increasing focus on its potential link with dyslipidemia. This study aims to investigate the relationship between dyslipidemia in expectant mothers and the risks of PTB. We studied 6963 mothers who gave birth at the International Peace Maternal and Child Health Hospital of Shanghai Jiaotong University School of Medicine in 2020, among which, 437 women had PTB. We extracted clinical and lipid data from electronic records, using multivariable logistic regression and restricted cubic spline models to explore the link between lipid concentrations (by quartiles) in pregnancy stages and PTB risk. The PTB rate was 6.3%. Early pregnancy in the PTB group showed elevated ApoA, ApoB, CHOL, LDL, and TG levels compared to controls (all P < 0.05). Late pregnancy showed no notable lipid differences. Multivariable analysis revealed elevated ApoA, TG, higher age, BMI ≥ 28 kg/m^2^, hypertension, assisted reproductive technology and gestational diabetes as PTB risk factors (all P < 0.05). After adjustments, higher ApoA, ApoB, CHOL and TG levels correlated with increased PTB risk. Using the lowest quartile, the adjusted ORs for early pregnancy's highest quartile of ApoA, ApoB, CHOL and TG were 1.348, 1.442, 1.442 and 2.156, respectively. Our findings indicate that dyslipemia in early pregnancy, including elevated levels of ApoA, ApoB, CHOL and TG, are associated with PTB. Managing lipid abnormalities during pregnancy may help reduce the risk of PTB.

## Introduction

Preterm birth (PTB) refers to a live infant’s delivery before completing 37 weeks of gestation, or within 259 days from the onset of a woman's last menstrual cycle^1–3^. It is concerning that 65% of PTB do not fit into any of the 21 identified risk categories, making it difficult to mitigate PTB risks^4^. Premature infants also exhibit differences in growth and immune response compared to full-term babies and account for 17.7% of under-five fatalities^5^. From a societal standpoint, PTB amplifies long-standing health expenditure and inflicts significant emotional and financial strain on families. Identifying PTB risk factors and establishing pertinent interventions is paramount.

There are two main types of PTB: spontaneous and iatrogenic PTB, with spontaneous PTB accounting for approximately 70% of cases^6,7^. There are various causes of PTB. Iatrogenic PTB can occur due to factors such as fetal distress, placental abruption, and multiple pregnancies^8^. On the other hand, spontaneous PTB can be triggered by factors like infection or inflammation, meconium-stained amniotic fluid, vascular disorders, and excessive stretching of the uterine cavity. Additionally, a history of previous PTB and cervical surgery can also contribute to the risk of spontaneous PTB^9^. While both clinical trials and retrospective studies have consistently linked past PTBs or multiple pregnancies to heightened PTB risks^10^, other risk determinants remain ambiguous.

Recent epidemiological evidence suggests that there are common risk factors between PTB and cardiovascular disease^11^. Women who experience PTB have a twofold higher risk of developing cardiovascular disease later in life compared to those who have full-term deliveries^12^. At least one third of placental biopsies from women with PTB present vascular lesions, with pathological changes including fibrinoid atherosclerosis, villi infarction, and thrombosis^13,14^. Dyslipidemia is a known and significant risk factor for vascular disease^15^. There is evidence that dyslipidemia is also associated with preeclampsia, a known risk factor for PTB^16^.

Our research aims to investigate the association between lipid levels, including ApoA, ApoB, CHOL, HDL, LDL, and TG, and PTB. These findings may provide guidance for the management of abnormal lipid levels during pregnancy, thereby reducing the incidence of PTB.

## Materials and methods

A retrospective study was conducted on 13,492 pregnant women who had regular obstetric check-ups and hospital deliveries at the International Peace Maternity and Child Health Hospital, Shanghai Jiao Tong University School of Medicine between January 1, 2020 and December 31, 2020. 586 (4.34%) cases of preterm delivery occurred in our cohort. The diagnostic criteria for preterm labor were referred to the Queensland Clinical Guidelines, 2020: Preterm Labor and Delivery. The inclusion criteria were singleton pregnancies with a live birth with a maternal age 18 years or older. The exclusion criteria were that there were no initial blood lipid level tests performed during in early (at 10–14 weeks gestation) and late pregnancy (at 28–32 weeks). Multiple pregnancies and pregnant women with a previous history of preterm delivery were also excluded^17^. Finally, a total of 6963 cases were included in this study, of which 437 (6.27%) cases had preterm deliveries (preterm delivery group) and 6526 cases delivered at term (control group).

This study followed the guidelines of the Declaration of Helsinki (6th revision, 2008). The informed consent was obtained from the participants and was approved by The Ethics Committee of the International Peace Maternity and Child Health Hospital ((GKLW)2020-03). As the data was anonymized, individual informed consent was not needed for this study. The study data were obtained from the hospital electronic medical record system (EMR system). General clinical data and blood lipid indices at 10–14 weeks of gestation were collected from the 2 groups of pregnant women. General clinical data included age at delivery, height, pre-pregnancy weight, pre-pregnancy body mass index (BMI), occupation, education, ethnicity, number of pregnancies, number of miscarriages, week of delivery, mode of delivery, mode of conception, history of smoking and alcohol, pregnancy comorbidities and past history. Lipid markers at 10–14 weeks and 28–32 weeks of gestation included apolipoprotein A (ApoA), apolipoprotein B (ApoB), cholesterol (CHOL), high density lipoprotein (HDL), low density lipoprotein (LDL) and triglycerides (TG). The lipid indexes in PTB and control groups were respectively categorized based on their quartiles (quartile 1: < 25thpercentile, quartile 2: 25th–50th percentile, quartile 3: 50th–75th percentile, quartile 4: > 75th percentile).

Normality of data was determined by using the Shapiro–Wilk test. Normally distributed quantitative data were expressed as x ± s, and t-test was used for comparative analysis. The rank sum test was used for continuous variables that were non-normally distributed. Qualitative data were expressed as frequencies (percentages), and rates were compared by using either chi-squared or Fisher’s exact tests. The multifactorial logistic regression model was used to analyze the relationships between blood lipid indexes and the risk of PTB at early and late gestation ages, respectively. In order to explore the shape of the curves obtained between PTB and lipid levels, the restricted cubic splines were used with knots at 3 in the finally adjusted models. SPSS version 25 software was used for statistical analysis of the study data. Two-sided tests were employed and P < 0.05 was considered statistically significant.

### Ethics approval and consent to participate

The informed consent was obtained from the participants and was approved by The Ethics Committee of the International Peace Maternity and Child Health Hospital ((GKLW)2020-03).

## Results

Among the 6963 pregnant women enrolled in this study, the prevalence of preterm delivery was 6.27%. The median age and quartiles of the PTB group were 32 (29, 35) years and the median pre-pregnancy BMI and quartiles of the PTB group were 21.2 (19.5, 23.4) kg/m^2^. Compared to the control group, the age and pre-pregnancy BMI of pregnant women in the PTB group were higher and showed significant differences in the mode of conception used (P < 0.05 in all cases). All the relevant data regarding our patients are shown in Table 1.

**Table 1:** Clinical maternal characteristics of the patients enrolled in this study.

	Control group (n = 6526)	PTB group (n = 437)	*P*
Age/[M(P 25, P 75), years old]	31 (29, 34)	32 (29, 35)	0.001
Hospital admissions/[M(P 25, P 75), times]	1 (1, 2)	1 (1, 2)	0.327
Gestational weeks/[M(P 25, P 75), week]	39.1 (38.4, 40)	35.5 (34.3, 36.3)	< 0.001
Gravida/[M(P 25, P 75), times]	1 (1, 2)	1 (1, 2)	0.014
Parity/[M(P 25, P 75), time]	1 (1, 2)	1 (1, 2)	0.375
Abortion/[M(P 25, P 75), times]	0 (0, 1)	0 (0, 1)	0.006
Pre-pregnancy BMI/[M(P 25, P 75), kg/m^2^]	20.9 (19.4, 23)	21.2 (19.5, 23.4)	0.186
Smoking/n (%)			0.235
Non-smoking	6505 (99.60%)	435 (99.50%)	
Smoking	21 (0.30%)	2 (0.50%)	
Alcohol drinking/n (%)			0.255
Non-drinking	6474 (99.20%)	433 (99.10%)	
Drinking	52 (0.80%)	4 (0.90%)	
Marriage/n (%)			0.700
Married	6493 (99.50%)	436 (99.80%)	
Unmarried	33 (0.50%)	1 (0.20%)	
Nation/n (%)			0.448
The Han nationality	6400 (98.10%)	432 (98.90%)	
Minority	112 (1.70%)	4 (0.90%)	
Foreign national	14 (0.20%)	1 (0.20%)	
Proportion of local patients/n(%)			0.104
Local	3368 (51.60%)	208 (47.60%)	
Ecdemic	3158 (48.40%)	229 (52.40%)	
Delivery mode/n (%)			< 0.001
Eutocia	3112 (47.69%)	165 (37.80%)	
Caesarean section	3035 (46.50%)	255 (58.40%)	
With the help of vacuum aspirators	28 (0.40%)	0 (0.00%)	
Low forceps	351 (5.40%)	17 (3.90%)	
Conception method/n (%)			0.014
Nature conceived	5218 (80.00%)	328 (75.10%)	
Unnatural conceived	1308 (20.00%)	109 (24.90%)	
Pre-pregnancy BMI/n(%)			0.032
< 18.5	763 (11.70%)	57 (13.00%)	
18.5–23.9	4764 (73.00%)	295 (67.50%)	
24–27.9	794 (12.20%)	63 (14.40%)	
> 28	202 (3.10%)	22 (5.00%)	
DM&GDM/n(%)			0.157
DM	32 (0.4%)	4 (0.9%)	
GDM	935 (14.3%)	109 (24.9%)	

The levels of ApoA, ApoB, CHOL, LDL and TG were higher in the PTB group compared with the control group in early pregnancy (all P < 0.05, Table 2). However, all the lipid levels in late gestation did not show any significant differences.

**Table 2:** Comparison of the blood lipid levels in early and late pregnancy between the preterm and control groups, respectively.

Index	Control group (n = 6526)	PTB group (n = 437)	*t* value	*P* value
Early pregnancy				
ApoA/(g L^−1^)	1.840 ± 0.326	1.890 ± 0.346	2.949	0.003
ApoB/(g L^−1^)	0.800 ± 0.144	0.810 ± 0.140	2.239	0.026
CHOL/(mmol L^−1^)	4.390 ± 0.729	4.490 ± 0.753	2.932	0.003
HDL/(mmol L^−1^)	1.870 ± 0.381	1.880 ± 0.403	0.521	0.602
LDL/(mmol L^−1^)	2.360 ± 0.589	2.420 ± 0.608	1.847	0.065
TG/(mmol L^−1^)	1.290 ± 0.557	1.400 ± 0.638	4.141	< 0.001
Late pregnancy				
CHOL/(mmol L^−1^)	6.420 ± 1.136	6.410 ± 1.126	-0.062	0.950
HDL/(mmol L^−1^)	2.020 ± 0.416	2.030 ± 0.442	0.206	0.836
LDL/(mmol L^−1^)	3.650 ± 1.057	3.630 ± 1.068	-0.451	0.652
TG/(mmol L^−1^)	3.240 ± 1.393	3.210 ± 1.336	-0.385	0.700

This study used crude and multifactorial logistic regression models to analyze the relationships between lipid indicators and the risk of PTB. The variables that showed significant variability in Table 1 were then included in a one-way logistic regression model. After that, a stepwise regression method was used to screen and the remove variables that caused multi-collinearity. It was found that all variables in the final regression model obtained were significant for the dependent variable. After hierarchical screening, the covariates were identified as age, pre-pregnancy BMI, hypertensive disorders gestational diabetes mellitus and assisted reproductive technology. The results (Table 3) suggested that ApoA,ApoB,TG and CHOL were risk factors for the development of PTB in early pregnancy (ORs and 95% CI for ApoA, ApoB, TG and CHOL were 1.584 (1.222–2.053), 2.481 (1.253–4.913), 1.456 (1.251–1.695)and 1.230 (1.078–1.403), respectively.), i.e., the risk of developing PTB gradually increased with the increase of the levels of these four indicators.

**Table 3:** Univariate and multivariate Logistic regression analysis of blood lipid levels in early pregnancy and the risk of PTB.

Variable	Control group/n	PTB group/n	Univariate Logistic regression analysis		Multivariate Logistic regression analysis	
			OR(95% CI)	*P* value	OR(95% CI)	*P* value
Early pregnancy						
ApoA/(g L^−1^)						
≤ 1.64	1734	89	ref		ref	
> 1.64 and ≤ 1.8	1572	109	1.351 (1.013–1.802)	0.041	1.351 (1.031–1.769)	0.029
> 1.8 and ≤ 1.97	1630	122	1.458 (1.101–1.932)	0.009	1.399 (0.992–1.974)	0.056
> 1.97	1590	117	1.434 (1.079–1.904)	0.013	1.348 (1.002–1.813)	0.048
ApoB/(g L^−1^)						
≤ 0.7	1735	98	ref		Ref	
> 0.7 and ≤ 0.79	1703	109	1.133 (0.856–1.501)	0.383	1.052 (0.775–1.428)	0.744
> 0.79and ≤ 0.88	1543	105	1.458 (0.907–1.600)	0.198	1.203 (0.883–1.638)	0.242
> 0.88	1545	125	1.434 (1.090–1.882)	0.01	1.442 (1.064–1.953)	0.018
CHOL/(mmol L^−1^)						
≤ 3.89	1678	99	ref		ref	
> 3.89 and ≤ 4.35	1641	107	1.105 (0.834–1.465)	0.486	1.132 (0.832–1.542)	0.43
> 4.35 and ≤ 4.85	1594	100	1.063 (0.799–1.416)	0.674	1.052 (0.767–1.443)	0.752
> 4.85	1606	131	1.383 (1.056–1.811)	0.019	1.442 (1.071–1.941)	0.016
HDL/(mmol L^−1^)						
≤ 1.61	1659	113	ref		ref	
> 1.61 and ≤ 1.86	1670	104	0.914 (0.695–1.204)	0.523	0.913 (0.675–1.235)	0.555
> 1.86 and ≤ 2.12	1618	102	0.926 (0.702–1.22)	0.583	0.916 (0.673–1.248)	0.58
> 2.12	1573	118	1.101 (0.843–1.438)	0.479	1.062 (0.786–1.437)	0.694
LDL/(mmol L^−1^)						
≤ 1.97	1675	99	ref		ref	
> 1.97 and ≤ 2.32	1608	108	1.136 (0.858–1.505)	0.373	1.002 (0.737–1.363)	0.989
> 2.32 and ≤ 2.72	1643	104	1.071 (0.807–1.422)	0.636	1.088 (0.801–1.477)	0.589
> 2.72	1594	126	1.337 (1.019–1.755)	0.036	1.284 (0.950–1.736)	0.104
TG/(mmol L^−1^)						
≤ 0.94	1695	72	ref		ref	
> 0.94 and ≤ 1.19	1646	111	1.588 (1.171–2.152)	0.003	1.730 (1.244–2.406)	0.001
> 1.19 and ≤ 1.52	1612	121	1.767 (1.31–2.384)	0	1.894 (1.364–2.632)	0
> 1.52	1567	131	1.968 (1.464–2.645)	0	2.156 (1.541–3.015)	0
Late pregnancy						
CHOL/(mmol L^−1^)						
≤ 5.62	1655	107	ref		ref	
> 5.62 and ≤ 6.34	1626	103	0.98 (0.741–1.295)	0.886	0.880 (0.645–1.200)	0.42
> 6.34 and ≤ 7.11	1631	123	1.166 (0.892–1.525)	0.26	1.153 (0.860–1.544)	0.341
> 7.11	1614	104	0.997 (0.754–1.317)	0.981	1.033 (0.762–1.399)	0.836
HDL/(mmol L^−1^)						
≤ 1.72	1630	116	ref		ref	
> 1.72 and ≤ 2	1665	110	0.928 (0.709–1.216)	0.589	0.952 (0.709–1.280)	0.746
> 2 and ≤ 2.29	1621	94	0.815 (0.616–1.079)	0.153	0.781 (0.571–1.068)	0.122
> 2.29	1594	116	1.023 (0.783–1.335)	0.87	1.021 (0.760–1.372)	0.89
LDL/(mmol L^−1^)						
≤ 2.93	1644	116	ref		ref	
> 2.93 and ≤ 3.57	1631	96	0.834 (0.631–1.103)	0.203	0.827 (0.606–1.128)	0.229
> 3.57 and ≤ 4.31	1625	116	1.012 (0.775–1.32)	0.932	1.014 (0.754–1.363)	0.927
> 4.31	1611	108	0.95 (0.725–1.246)	0.711	0.990 (0.734–1.334)	0.945
TG/(mmol L^−1^)						
≤ 2.34	1668	106	ref		ref	
> 2.34 and ≤ 2.93	1604	106	1.04 (0.788–1.373)	0.783	1.015 (0.751–1.371)	0.925
> 2.93 and ≤ 3.77	1631	121	1.167 (0.892–1.529)	0.26	1.159 (0.865–1.552)	0.324
> 3.77	1622	103	0.999 (0.755–1.322)	0.996	1.077 (0.792–1.465)	0.635

**Figure 1:**
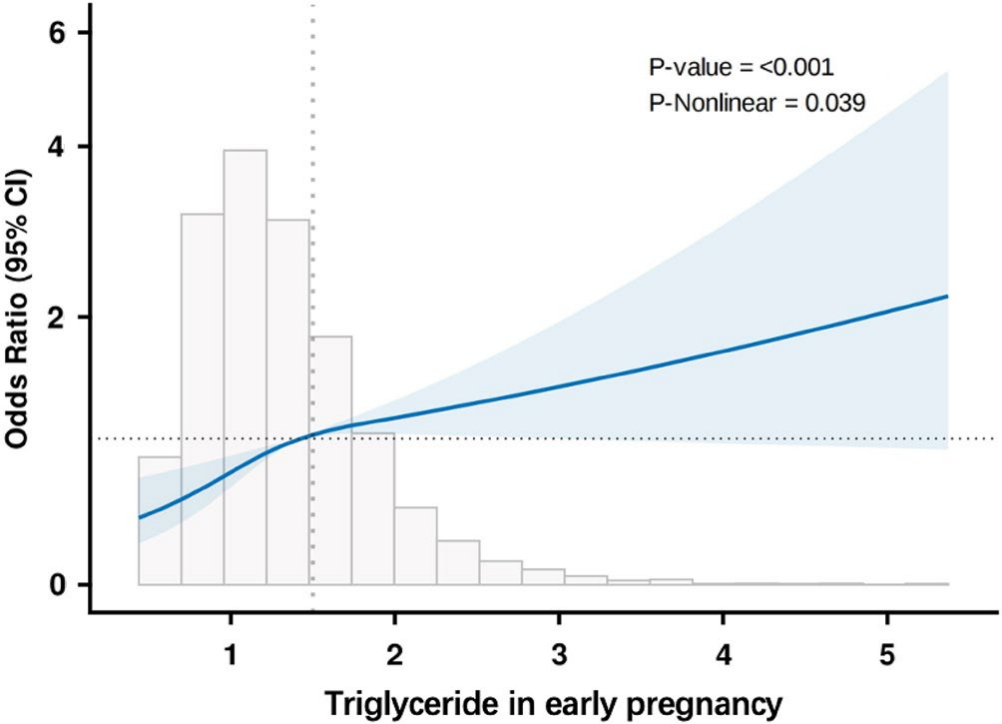
The association between triglyceride levels in early pregnancy and PTB by using the RCS function. The model had 3 knots located at the 10th, 50th and 90th percentiles. The y-axis represents the OR to present PTB for any value of triglycerides in early pregnancy compared to individuals with 1.5 of triglycerides in early pregnancy. The logistic regression was adjusted for hypertension disease, gestational diabetes mellitus (GDM), BMI, ART and age.

To further investigate the relationship between ApoA, ApoB, CHOL, TG and PTB, firstly, a non-linear test was performed for each of these parameters, in which the p-non-linear between ApoA, ApoB, CHOL and risk of PTB was > 0.05. This was performed to indicate that the non-linear relationship between them was not statistically significant (Supplementary Fig. 1). In contrast, the P-non-linear < 0.05 between TG and risk of PTB indicated a non-linear relationship, and therefore the relationship between TG and risk of PTB was visualized using a restricted cubic spline plot as shown in Fig. 1. The graph depicts an overall upward trajectory for the curve, with a notable change in slope occurring around a TG value of 1.5 mmol/L. When TG is below 1.5 mmol/L, the association between TG and PTB appears more subdued. However, when TG exceeds 4.25 mmol/L, there’s a heightened risk of PTB.

## Discussion

We reviewed 6963 pregnant women, 437 of whom had PTB (6.27%). We analyzed lipid levels in early and late pregnancy using multiple logistic regression and restricted cubic splines, and found that lipid levels have an effect on PTB.

In early pregnancy, PTB group showed higher levels of ApoA, ApoB, CHOL, LDL and TG than controls—a finding consistent with most previous research. Contrarily, the levels of each lipid species in late pregnancy did not show any statistically significant differences. Historically, continuous lipid indicators have been directly used to gauge PTB risk^18^. But, considering lipid indicator fluctuations across ethnicities, regions, and hospitals^19^, our study assessed the hierarchical effect (quartiles) between lipid levels and PTB risk. In univariate logistic regression, ApoA > 1.64 g/L, ApoB > 0.88 g/L, CHOL > 4.85 mmol/L, LDL > 2.72 mmol/L, TG > 0.94 mmol/L was statistically significant (p < 0.05). After adjustments for pre-pregnancy BMI, age at delivery, hypertensive disorders, gestational diabetes and conception method, our study discerned ApoA, ApoB, CHOL and TG lipid profiles in early pregnancy as independent risk factors for the development of PTB. In the contrast, HDL and LDL showed no strong correlation. In the analysis of lipid indicators in early pregnancy, the non-linear relationship between ApoA, ApoB, CHOL and risk of PTB was not statistically significant, but a trend towards their positive association was evident from the graph (Supplementary Fig. 1). The normal range for TG is around 0.56–1.70 mmol/L. Generally speaking, triglyceride levels do not physiologically rise too much in pregnant women in early pregnancy. After visualizing the relationship between TG and the risk of PTB (p-nonlinear = 0.039), it can be seen that when TG are less than 1.5 mmol/L (Fig. 1), the risk of PTB increases significantly with increasing TG levels; in other words, the risk of PTB decreases dramatically for every unit of decrease in TG. When TG were greater than 1.5 mmol/L, the increase in the risk of PTB became more modest as TG levels increased. Although the slope between TG and the risk of PTB changed before and after 1.5 mmol/L, the overall trend of the curve was positive, making it essential to encourage pregnant women to actively manage their TG levels, even if they are within the normal range. For example, those with excessive levels should be encouraged to actively adjust their diet and exercise; those with normal levels should be encouraged to maintain their original lipid levels.

Pregnancy is marked by dynamic lipid fluctuations^20^. From the 12th week of gestation, elevated levels of CHOL, LDL, HDL and TG emerge due to estrogen stimulation and insulin resistance^21^. This metabolic shift is crucial for fetal growth and provides energy for the mother^22^. However, emerging research suggests that abnormal lipid levels during pregnancy may be associated with the onset of preterm labor^23^. A systematic review and review the relationship between lipid levels and PTB, found that different levels of TC, TG, HDL, LDL and Homocysteine (Hct) have different degrees of influence on the occurrence of PTB^24^. And in a prospective cohort study conducted by Sharami et al., 378 pregnant women from the Al-Zahra Hospital were randomly selected to investigate their levels of total cholesterol (TC), HDL, LDL and TG. The results showed that among the lipid profiles evaluated, the predictive power of TG for PTB had an AUC = 0.833 (95%, CI 0.736–0.930), respectively. In addition, abnormal LDL concentrations increased the risk of PTB by twofold (P < 0.05)^25^.

Our results found that the higher the level of AopA, the greater the risk of PTB. Although the third quantile was not statistically significant, OR still appeared as a risk factor for PTB (OR = 1.399). ApoA is a protein in the blood that is a key component of HDL. It helps transport cholesterol from tissues to the liver for removal, playing a role in preventing cardiovascular diseases like atherosclerosis. However, previous studies have also shown that AopA may also negatively affect endothelial progenitor cell (EPC) -induced angiogenesis by inhibiting EPC proliferation, adhesion, migration, and angiogenesis^26^. Although ApoA is a major component of HDL, our study did not find a link between HDL and PTB. Zvintzou et al. may provide some references. Through experiments, they found that transgenic mice that overexpress APOA-II can cause changes in other components of HDL, thereby affecting its reverse transport and protective effect on cholesterol. In addition, high levels of APOA-II may also affect physiological processes such as vascular function and inflammatory response, and promote the occurrence of atherosclerosis^27^. Hardening of the arteries can affect the microcirculation in a pregnant woman, leading to a lack of blood perfusion in the placenta, which increases the risk of PTB^28^. ApoB is mainly found in LDL and Very low-density Lipoprotein (VLDL) and is an important component of cholesterol metabolism. And High ApoB is a risk factor for coronary heart disease and arteriosclerosis, which may be a link between these and PTB.

CHOL and TG are common blood lipids that are essential for human health. CHOL plays a role in cell membrane structure, hormone synthesis, and nerve conduction, but high levels of LDL cholesterol may increase atherosclerosis risk^29^. TG is a major form of fat storage and source of energy, but high levels may be linked to metabolic syndrome and cardiovascular disease^30^. Recent studies suggest that higher TG during pregnancy could elevate the risk of preeclampsia and preterm delivery^22^. Catov et al. conducted a nested case control study with 300 individuals and corrected for gestational age, BMI and ethnicity at the time of sampling for lipid concentrations which was before 15 weeks of gestation. They concluded that elevated CHOL and TG levels in early gestation were associated with a 2.8-fold and 2-fold increased preterm delivery risk before 34 weeks and between 34 and 37 weeks, respectively^31^. They also suggested that lipid changes could relate to inflammation and infection, and that hypertriglyceridemia may be considered part of the innate immunity and that increased inflammatory proteins may cause hypercholesterolemia. In addition, studies have shown that high cholesterol may contribute to the formation of blood clots, which can lead to complications during pregnancy, such as placental abruption, which can promote PTB^32^.

Currently, most research focuses on the first trimester^33–35^. Our study, which included lipid indicators in the first and third trimester, found a significant association between lipid levels in the first trimester and the risk of preterm birth, as opposed to the lack of association in the third trimester, which also raises an important question about the underlying mechanism. One possible explanation may have to do with key developmental processes that occur in early pregnancy, including placenta formation and embryonic organogenesis^36–38^. It is known that disturbances in lipid metabolism during this sensitive period can disrupt these key processes, leading to adverse pregnancy outcomes such as preterm birth^39^. In addition, metabolic and hormonal changes in early pregnancy may make the mother and fetus more vulnerable to abnormal lipid levels^40^. Together, these factors contribute to the observed association between blood lipid levels and preterm birth, especially in the first trimester.

Although our study's data stemmed from real-case scenarios and attempted to mitigate biases, certain limitations persist. Some include potential biases from missing data, single-center study constraints, and absence of preterm delivery type classifications. In addition, some of the population characteristics that did not show statistically significant differences in clinical practice may still be strongly correlated with PTB, such as the mother's education level, the number of births experienced, etc. Since these variables was not corrected for, it may have caused some bias to the results of the multifactorial regression^41^. Future multi-center studies must consider refining confounding variables and incorporating more clinical variables to validate our findings.

## Conclusion

In summation, our findings underscore that dyslipemia in early pregnancy augments the risk of PTB. Evaluating lipid markers in this crucial phase can preemptively identify expectant mothers with heightened PTB vulnerabilities, enabling timely clinical interventions. It is crucial for healthcare providers to carefully monitor individuals displaying lipid irregularities in the early stages of pregnancy and to continue tracking changes in lipid levels as the pregnancy advances. This vigilant approach allows for the early detection and management of potential complications, highlighting the importance of lipid monitoring in prenatal care to ensure the health and well-being of both the mother and the developing fetus.

### Supplementary Information

Supplementary Figure 1.

## Data Availability

The datasets generated and/or analysed during the current study are not publicly available due to ethical reason but are available from the corresponding author on reasonable request.

## Abbreviations

PTB: Preterm birth
ApoB: Apolipoprotein B
ApoA: Apolipoprotein A
TG: Triglyceride
CHOL: Cholesterol
HDL: High density lipoprotein
LDL: Low density lipoprotein
EMR system: Electronic medical record system
BMI: Body Mass Index
GDM: Gestational diabetes mellitus
