# Supplementary material for: The association between pregnancy levels of blood lipids and the risk of preterm birth
Source: Sci Rep. 2024 May 11;14:10800. doi: 10.1038/s41598-024-61119-x (PMC11088646; doi:10.1038/s41598-024-61119-x)
Supplement: Supplementary file 1 — Supplementary Figure 1. [file 41598_2024_61119_MOESM1_ESM.pdf]

The association between pregnancy levels of blood lipids and the risk of preterm birth

Yao Lv[1,2,3*], Liang Xu[1,2,3*], Zhong He[4], Xiaorui Liu[1,2,3#], Yuna Guo[1,2,3#]

*co-first author. These authors contributed equally to this work

#co-corresponding authors.

[1]The International Peace Maternity and Child Health Hospital, School of Medicine, Shanghai Jiao Tong University, Shanghai, China

[2]Shanghai Key Laboratory of Embryo Original Diseases, Shanghai, China

[3]Shanghai Municipal Key Clinical Specialty, Shanghai, China

[4]Songjiang Maternity and Child Health Hospital, Shanghai, China

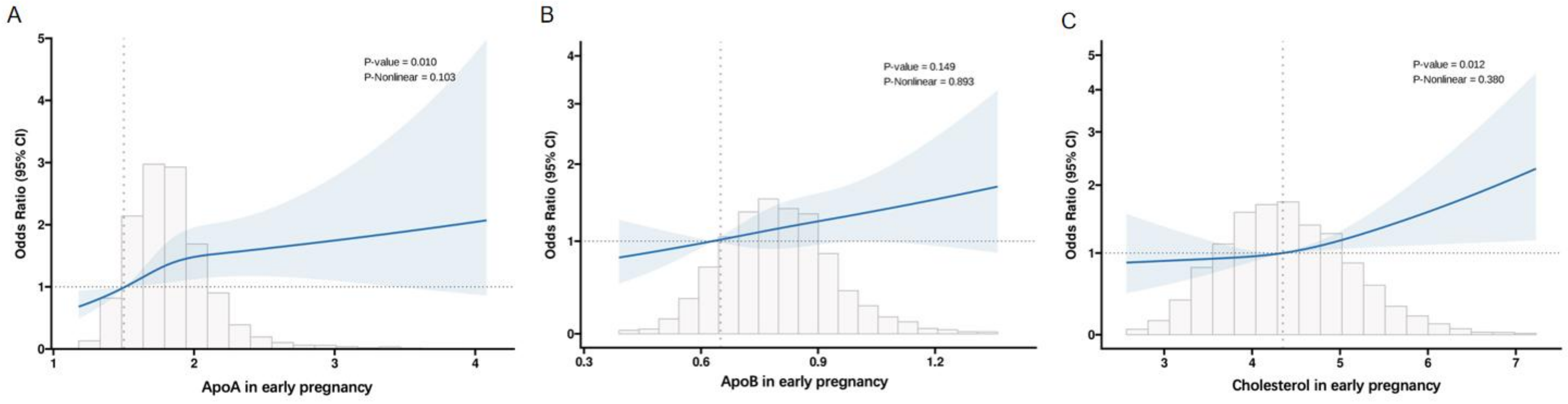

Supplementary Figure 1A. The association between ApoA in early pregnancy and PTB by using the RCS function. The model had 3 knots located at the 10th, 50th and 90th percentiles. The y-axis represents the OR to present the PTB for any value of ApoA in early pregnancy compared to individuals with 1.501 of ApoA in early pregnancy. The logistic regression was adjusted for hypertension disease, GDM, BMI, ART and age. 1B. The association between ApoB in early pregnancy and PTB by using the RCS function. The model had 3 knots located at the 10th, 50th and 90th percentiles. The y-axis represents the OR to present the PTB for any value of ApoB in early pregnancy compared to individuals with 0.65 of ApoB in early pregnancy. The logistic regression was adjusted for hypertension disease, GDM, BMI, ART and age. 1C. The association between CHOL in early pregnancy and PTB by using the RCS function. The model had 3 knots located at the 10th, 50th and 90th percentiles. The y-axis represents the OR to present the PTB for any value of CHOL in early pregnancy compared to individuals with 4.35 of CHOL in early pregnancy.The logistic regression was adjusted for hypertension disease, GDM, BMI, ART and age.
